# Evolving liver inflammation in biochemically normal individuals with anti-mitochondria antibodies

**DOI:** 10.1186/s13317-019-0120-x

**Published:** 2019-10-31

**Authors:** Danielle Cristiane Baldo, Alessandra Dellavance, Maria Lucia Gomes Ferraz, Luis Eduardo C. Andrade

**Affiliations:** 1 Rheumatology Division, Universidade Federal de São Paulo, UNIFESP, Rua Botucatu 740, São Paulo, SP 04023-900 Brazil; 2 Research and Development Division, Fleury Medicine and Health Laboratories, São Paulo, Brazil; 3 Gastroenterology Division, Universidade Federal de São Paulo, UNIFESP, São Paulo, Brazil

**Keywords:** Autoimmune liver diseases, Primary biliary cholangitis, Pre-autoimmunity, Disease prevention, ELF score

## Abstract

**Background:**

Anti-mitochondria autoantibodies (AMA) occur in > 95% primary biliary cholangitis (PBC) patients. Biochemically normal AMA-positive (BN/AMA+) individuals, occasionally noticed by indirect immunofluorescence (IIF) on HEp-2 cells and confirmed in AMA-specific assays, may represent early stages of PBC. The Enhanced Liver Fibrosis (ELF) score is a surrogate marker for liver fibrosis. This prospective study investigated the ELF score in BN/AMA+ individuals and PBC patients, considering autoantibody avidity and serum levels along the years.

**Methods:**

327 samples from 35 PBC and 59 BN/AMA+ were prospectively obtained in average 3.83 (range 0.50–7.40) years apart. Samples were tested by IIF on rat-kidney (IIF-AMA), western-blot for AMA (WB-AMA), and ELISA for antibodies against pyruvate-dehydrogenase (PDC-E2), gp210, sp100 and CENP-A/B. Anti-PDC-E2 avidity was determined by 6 M urea-elution ELISA. Alkaline phosphatase (ALP), gamma glutamyl transferase (ɣGT) and ELF score were measured by automated methods.

**Results:**

Along the follow-up period BN/AMA+ subjects and PBC patients presented significant increase in serum anti-PDC-E2 (mean 10.45% and 8.86% per year; respectively), anti-PDC-E2 avidity (3.02% and 4.94%/year) and ELF score (3.24% and 2.71%/year). IIF-AMA and ɣGT increased in BN/AMA+ (6.59% and 2.36%) and decreased in PBC (− 4.89%/year and − 3.88%/year). In BN/AMA+ individuals there was positive correlation of ELF with IIF-AMA titer (r = 0.465; p < 0.001) and with anti-PDC-E2 levels (r = 0.239; p < 0.001). Expansion of autoantibody targets along time occurred in 39% BN/AMA+ and 49% PBC patients. The frequency of BN/AMA+ with high probability of having established PBC increased from 7 to 14%.

**Conclusions:**

BN/AMA+ individuals present an orchestrated increase in ELF score and humoral autoimmune response over time, indicating an opportunity for early therapeutic intervention and prevention in autoimmunity.

## Background

Circulating autoantibodies precede the clinical onset of several autoimmune diseases [1–4]. In this respect, primary biliary cholangitis (PBC) is of special interest because its serologic signature [anti-mitochondrial antibodies (AMA)] precedes the development of biochemical abnormalities and clinical manifestations by years or decades [5, 6]. Median survival in untreated patients was traditionally considered to be 7.5 to 16 years [7], but has been vastly improved by the introduction of ursodeoxycholic acid (UDCA) therapy. Patients treated with UDCA at early stages of the disease tend to respond well to therapy and can reach life expectancy equivalent to the general population [8–15].

Others and we have recently shown that the humoral autoimmune response in the preclinical and pre-biochemical (normal liver enzymes) phase of PBC is less intense than that observed in patients with established PBC [16–18]. In a previous study, we showed that high titer AMA, high avidity anti-pyruvate dehydrogenase complex E2 subunit (PDC-E2) antibodies, and recognition of multiple cell domains by autoantibodies represent risk factors for a given AMA-positive subject to develop established PBC. Dahlqvist et al. also observed higher titer AMA and higher frequency of PBC-specific antinuclear antibodies (ANA) to be associated with established PBC [17]. The temporal dynamics of increased autoimmune responses during the pre-clinical/pre-biochemical phase of PBC has not been investigated.

Liver histology has not been thoroughly studied in biochemically normal AMA-positive (BN/AMA-positive) individuals. This is due in part to ethical constraints in obtaining biopsy liver fragments from BN/AMA-positive individuals. The recent development of biochemical markers for the assessment of liver fibrosis [19–22] offers the possibility of investigating the inflammatory and fibrotic status of the liver in BN/AMA-positive individuals. One particularly powerful non-invasive assessment of liver histology is the Enhanced Liver Fibrosis (ELF) score, an algorithm capable of estimating the degree of hepatic fibrosis based on the serum concentrations of the tissue inhibitor of metallo-proteinases-1 (TIMP-1), amino-terminal propeptide of type III procollagen (PIIINP) and hyaluronic acid (HA) [19]. The consistent performance of the ELF score in the assessment of liver fibrosis in various diseases, including PBC [20–22], offers a remarkable opportunity to investigate the hepatic status of BN/AMA-positive individuals and perhaps to identify which of these individuals will go on to develop PBC. The ability to recognize and diagnose patients at early stages of PBC natural history would offer an opportunity for very early therapeutic intervention with UDCA. In this study, we performed a prospective temporal analysis of humoral autoimmune response and biochemical indicators of hepatic injury in BN/AMA-positive individuals and PBC patients.

## Methods

This prospective temporal analysis aimed to investigate evidence of liver fibrosis or inflammation in a cohort of BN/AMA-positive individuals. In addition, we documented the temporal evolution of intrinsic features of the humoral autoimmune response (avidity and serum level of autoantibodies; spectrum of autoantibodies) in BN/AMA-positive individuals in relation to the ELF score, a biochemical parameter of liver fibrosis.

### Subjects and samples

Subjects were retrieved from among individuals that participated in our previous study [16] and were chosen based on two criteria: (1) suspicion of AMA based on characteristic mitochondria-like speckled cytoplasmic staining pattern in the indirect immunofluorescence assay (IFA) on HEp-2 cells (HEp-2 IFA); and (2) AMA confirmation by specific assays (indirect immunofluorescence on rodent tissue, ELISA, and Western blot). Details on the methodology of these tests are provided in our previous study [16] and below. PBC diagnosis was established according to the criteria of the European Association for the Study of the Liver [23]. BN/AMA-positive individuals had no apparent disease and presented normal alkaline phosphatase (ALP) serum levels (at least 6 months within the date of evaluation). Clinical data were obtained by chart review or interview with the physicians who ordered the tests. Sequential samples were retrieved every time subjects had laboratory tests ordered by their physicians in the period of August 2005 to July 2014. Autoantibodies and serum liver enzymes were determined in most samples. In this period, we obtained serial samples (with at least 6 months between samplings) from 35 PBC patients and 64 BN/AMA-positive individuals. Twenty-five PBC patients (71%) were using UDCA. Five BN/AMA-positive individuals started UDCA therapy during the study period and were therefore excluded, leaving 59 BN/AMA-positive individuals in the study.

### Temporal prospective analysis

Sequential samples were retrieved when individuals returned to the laboratory for further tests. The number of samples and the time interval between sequential samples were heterogeneous. To cope with this heterogeneity, we grouped data into what we have termed sequential “time stations” separated by 1 year. The baseline sample was designated time station T_0_. Each individual had one sample at T_0_ and at least one sequential sample at a subsequent time station, and there was no more than one sample per individual per time station. The total number of samples in each time station varied, as shown in Table 1. This arrangement was used for describing data in some graphs, but the actual statistical analysis was performed with primary time data (see ahead).

**Table 1:** Number of samples in BN/AMA+ individuals and PBC patients according to time stations and respective time intervals

Time stations	Interval from baseline (years)		Number of individuals in each group	
	Minimum	Maximum	BN/AMA+	PBC
T_0_	–	–	59 (100%)	35 (100%)
T_1_	≥ 0.5	< 1.5	23 (39%)	9 (26%)
T_2_	≥ 1.5	< 2.5	20 (34%)	12 (34%)
T_3_	≥ 2.5	< 3.5	26 (44%)	14 (40%)
T_4_	≥ 3.5	< 4.5	26 (44%)	17 (49%)
T_5_	≥ 4.5	< 5.5	21 (36%)	14 (40%)
T_6_	≥ 5.5	< 6.5	16 (27%)	17 (49%)
T_7_	≥ 6.5	< 7.5	8 (14%)	10 (29%)

*BN/AMA+* biochemically normal and anti-mitochondria antibody-positive individuals, *PBC* primary biliary cholangitis

Volume limitation prevented us from carrying out all tests in some samples. Therefore, there is some heterogeneity in the number of samples tested for each biochemical and immunological parameter along the time stations.

### Detection of autoantibodies by indirect immunofluorescence assays (IIF)

IIF for anti-mitochondria antibodies (IIF-AMA) was performed on rodent tissue cryo-sections prepared in-house as described elsewhere [24]. Samples were screened at 1:40 in phosphate-buffered saline pH 7.4 (PBS) and serially diluted to end-point fluorescence to a limit of 1:2560. AMA reactivity was assessed with fluorescein isothiocyanate (FITC)-conjugated goat anti-human IgG antibodies (Biomérieux, France) at 1:200 in PBS. Samples were considered to be positive for IIF-AMA if the characteristic mitochondria-like pattern was observed in kidney tubular cells and hepatocytes.

The HEp-2 IFA test was performed using a 1:80 dilution of subject sample on HEp-2 cell slides (MBL-Bion Enterprise Ltd, USA) following the manufacturer’s instructions. In addition to the speckled cytoplasmic staining pattern indicating reaction with mitochondria, we also recorded reactivity to the nuclear envelope, multiple nuclear dots, and centromeres. Slides were independently analyzed by two blinded readers (AD and DCB) using an Olympus (Japan) B50 fluorescence microscope at ×400 magnification.

### Detection of anti-PDC-E2 IgG by ELISA

An in-house ELISA assay for anti-PDC-E2 was established as previously described [16, 25]. Briefly, Nunc Maxisorp plates (Thermo Fisher Scientific, USA) were coated with 100 μL PDC-E2 from porcine heart (Sigma-Aldrich, USA) at 10 μg/mL in 0.1 M carbonate-bicarbonate buffer pH9.6 at 4 °C overnight. Plates were washed three times in PBS containing 0.05% Tween (Sigma-Aldrich, USA) (PBS-T) and individual wells were then incubated with 100 μL serum at a 1:400 dilution in 0.05% Tween, 0.5% bovine serum albumin (BSA) in PBS (PBS-BT) for 1 h at 37 °C. After washing as before, wells were incubated with peroxidase-labeled goat anti-human IgG (γ-chain specific) antibody (Sigma-Aldrich, USA) diluted 1:20,000 in PBS-BT at 37 °C for 1 h. After washing as before, horseradish peroxidase (HRP) enzyme activity was detected with 100 μL 3,3′,5,5′-tetramethylbenzidine (TMB) with hydrogen peroxide (Siemens, Germany) for 20 min at room temperature. The reaction was stopped by adding 100 μL of 4 N sulfuric acid, and the resulting yellow color was measured at 450 nm in a spectrophotometer VICTOR™ X3 (PerkinElmer, USA). High- and low-reactivity standards were obtained from INOVA Diagnostics (USA). The cut-off was established as four times the absorbance of a series of negative samples from the laboratory staff. Reactivity was expressed as arbitrary units (AU) calculated as the ratio of the optical density (OD) of the sample over the cut-off level. Samples with reactivity above 1.0 AU were considered reagent.

### Determination of anti-PDC-E2 antibody avidity

The avidity of anti-PDC-E2 IgG was determined by elution under chaotropic conditions [16]. Samples were incubated in quadruplicate in the standard anti-PDC-E2 ELISA plate for 1 h. For each quadruplicate set, two wells were incubated with regular washing solution and two wells were incubated with 6 M urea in PBS-T for 15 min at room temperature. Plates were then washed in regular PBS-T and further processed as per the regular ELISA. Avidity was estimated by dividing the optical density at 450 mn observed in the wells submitted to urea treatment by the optical density in wells without urea treatment.

### Detection of anti-mitochondria antibodies by Western blot

IgG reactivity against E2 subunits of the 2-oxoacid dehydrogenase complex (2-OADC), including the PDC-E2—74 kDa, Branched-chain 2-Oxo-acid Dehydrogenase (BCOADC—56 kDa), 2-Oxo-glutarate Dehydrogenase (OGDC—52 kDa), and the E3 Binding Protein of dihydrolipoamide dehydrogenase (36 kDa), was determined by Western blot (WB) as described previously [26–28]. Briefly, mitochondria-rich liver extract (10 mg/mL) was separated by 10% sodium dodecyl sulfate-polyacrylamide gel electrophoresis and transferred onto nitrocellulose filters using iBlot^®^ System (Invitrogen, USA). After blocking in PBS containing 5% skim milk (PBS-M) for 2 h, longitudinal strips were incubated with individual serum samples diluted 1:50 in PBS-M for 1 h. Strips were then washed three times in PBS-T and incubated for 1 h at room temperature in HRP-conjugated rabbit anti-human IgG (Bio-Rad, USA) 1:1500 in PBS-M. The colorimetric reaction was developed for 10 min in 6 mg 4-chloro-1-naphtol (Thermo Fisher Scientific, USA) diluted in 2 mL methanol (Merck, Germany) added to 10 mL PBS containing 20 µL 30% H_2_O_2_. The reaction was stopped with distilled water after the development of bands.

### Detection of antibodies to nuclear envelope protein gp210, nuclear protein sp100 and centromere proteins (CENP-A and CENP-B) by ELISA

Reactivity at 1:101 serum dilution to purified gp210 and sp100, and to recombinant CENP-A/B was determined by ELISA (INOVA Diagnostics, USA), according to the manufacturer’s instructions. These ELISA assays were performed only on samples that showed nuclear envelope, multiple nuclear dots, or centromere staining patterns in the HEp-2 IFA test in at least one of the serial samples.

### Analysis of the range of targets of the humoral autoimmune response

We analyzed general autoimmune response using IIF. We further analyzed the range of specific targets of the humoral autoimmune response using WB (mitochondrial proteins 74 KDa, 56 KDa, 52 KDa and 48 KDa) and ELISA (anti-PDC-E2, anti-sp100, anti-gp210 and anti-CENP-A/B). All study subjects had AMA detectable by one or more of the three assay methods. In order not to be redundant, for this analysis we consider the ELISA assay for anti-PDC-E2 and the recognition of the protein of 74 kDa by WB as referring to the same target.

### PBC-like humoral autoimmune profile

In a multivariate analysis, we previously established that three variables were independently and strongly associated with the probability of any given AMA-positive sample belong to a patient with established PBC: high-titer AMA, high-avidity anti-PDC-E2 antibodies, and the occurrence of three or more PBC-specific antibodies [16]. These three variables were used to derive a model to define the strength of association with established PBC using a cohort of 151 BN/AMA-positive individuals and 61 patients with established PBC [16]. According to this model, the strength of association of the humoral autoimmune profile with PBC was defined as Weak (< 36%), Medium (36–72%) and Strong (> 72%). This model was then applied to classify serum samples in the present cohort.

### Biochemical biomarkers

ALP (female: 35–104U, male: 40–129U) and gamma-glutamyl transferase (ɣGT; female < 41U, male < 73U) were determined by kinetic colorimetric method on Cobas^®^ 8000 analyzer (F. Hoffmann-La Roche Ltd, Switzerland) using standard methods.

ELF score is calculated by an algorithm combining the serum concentration of TIMP-1, PIIINP and HA as follows: ELF score = 2.494 + 0.846 ln(C_HA_) + 0.735 ln(C_PIIINP_) + 0.391 ln(C_TIMP1_). These analytes were determined using an ADVIA Centaur CP immunochemical analyzer (Siemens, Germany) according to the manufacturer’s instructions. According to the literature, the indication of the degree of fibrosis of ELF score is as follows: None to mild (< 7.7); Moderate (7.7–9.8); Severe (> 9.8) [19].

### Statistical analysis

To compare groups in relation to gender, we used the Chi-square test. To compare age, number of samples and intervals between samples we used the Student’s t test (parametric variables) or Mann–Whitney’s test (non-parametric variables). The statistical inference for the relative delta (difference between any time point station and baseline value divided by baseline value) was calculated using ANOVA for repeated measures. The associations between group and the laboratory variables ɣGT and IIF-AMA were analyzed using simple generalized linear mixed model with logarithmic link function and Poisson distribution—due to the data type of ɣGT and IIF-AMA (integer data). The effect of time and its interaction with group were included. For all the other laboratory variables (continuous positive data), simple generalized linear mixed model with gamma distribution was used instead. Spearman’s correlation coefficient was used to calculate the correlation between variables. Wilcoxon’s test was used to compare the number of targets and changes in PBC-like humoral autoimmune profile in paired samples. The analysis was developed using glmer function from lme4 package of the software R v. 3.4.4 and IBM (USA) SPSS Statistics v20. A p value below 0.05 was considered significant.

## Results

PBC and BN/AMA-positive groups did not differ with regard to gender (85.7% female vs 94.9% female), age [54.7 ± 13.67 years (30–83) vs 50.7 ± 13.70 years (19–83)] or number of collected samples [total of 128 with average of 3 samples per individual (2–6) vs total of 199 with average of 3 samples per individual (2–6)]. The 327 samples from PBC patients and BN/AMA-positive individuals were obtained along an average time interval of 4.1 years (range 0.5–7.1) and 3.6 (range 0.5–7.4) years, respectively.

### AMA and biochemical markers are higher in PBC patients

At baseline, PBC patients had significantly higher anti-PDC-E2 serum levels (mean 2.9 ± 1.9 AU vs 2.0 ± 1.6 AU; p = 0.044), anti-PDC-E2 avidity (68% ± 21% vs 61% ± 24%; p = 0.014) and IIF-AMA (median 1/1280 (1/40–1/20,480) vs 1/320 (1/40–1/20,480); p < 0.001) compared to BN/AMA-positive individuals, confirming our previous results [16]. We also found that PBC patients had significantly higher serum ALP (200.4 ± 167.9 vs 69.2 ± 30.8; p < 0.001), and ɣGT (226.8 ± 303.4 vs 35.3 ± 46.8; p < 0.001), but not ELF score (8.6 ± 1.6 vs 7.9 ± 1.1; p = 0.169) (Table 2).

**Table 2:** Descriptive analysis of biochemical and autoantibody parameters in successive time stations in BN/AMA+ individuals and PBC patients

	Parameters					
	ALP (U/L)^a^	ɣGT (U/L)^a^	ELF score^a^	Anti-PDC-E2 (AU)^a^	Avidity anti-PDC-E2 (%)^a^	IIF-AMA (titer)^b^
BN/AMA+ individuals						
T_0_	69.2 (27–158)	35.3 (3–265)	7.9 (6–9.9)	2.0 (0.1–6.0)	61 (16–118)	320 (40–20,480)
T_1_	71.3 (28–105)	45.2 (8–191)	8.4 (6.4–10.5)	1.9 (0.3–6.4)	54 (Ø–105)	640 (Ø–10,240)
T_2_	63.4 (30–127)	33.2 (6–148)	8.8 (7.3–10.5)	2.4 (0.2–5.9)	55 (Ø–101)	160 (Ø–10,240)
T_3_	62.2 (31–131)	31.2 (4–179)	8.8 (7.2–10.7)	2.4 (0.2–5.9)	70 (33–99)	640 (Ø–20,480)
T_4_	63.0 (38–79)	25.2 (7–99)	9.2 (7.4–10.8)	2.9 (0.2–7.0)	62 (Ø–101)	480 (Ø–10,240)
T_5_	72.0 (42–198)	31.9 (10–111)	8.7 (5.8–10.2)	2.4 (0.2–7.4)	63 (Ø–97)	320 (Ø–10,240)
T_6_	79.0 (42–126)	39.8 (7–82)	9.3 (8.5–11.2)	3.9 (0.2–7.7)	88 (5–121)	640 (160–20480)
T_7_	52.8 (37–67)	82.2 (12–308)	9.2 (9–9.5)	2.5 (0.3–4.9)	64 (Ø–93)	160 (Ø–2560)
PBC patients						
T_0_	200.4 (52–776)	226.8 (10–1221)	8.6 (5.8–13.5)	2.9 (0.2–7.2)	68 (26–105)	1280 (40–20,480)
T_1_	138.5 (77–360)	145.6 (15–524)	9.1 (7–10.6)	3.4 (0.4–7.4)	71 (Ø–97)	1280 (Ø–5120)
T_2_	86.7 (35–128)	95.6 (19–341)	9.1 (6.9–11.4)	3.4 (0.4–6.7)	69 (Ø–96)	480 (Ø–5120)
T_3_	138.7 (46–490)	105.7 (10–307)	9 (4.7–12.5)	3.6 (0.4–7.8)	77 (Ø–112)	1280 (80–20,480)
T_4_	130.6 (55–259)	144.1 (13–473)	9.7 (7.8–13.4)	3.4 (0.3–6.4)	77 (Ø–104)	1280 (Ø–10,240)
T_5_	311.6 (66–518)	387.8 (10–1545)	10.6 (9.1–12.1)	5.1 (1.7–7.4)	81 (3–105)	1280 (40–10,240)
T_6_	167.3 (65–359)	138.6 (13–579)	9.7 (8.4–12)	4.3 (0.3–6.7)	90 (59–107)	1280 (320–10,240)
T_7_	190.0 (110–527)	258.4 (21–1448)	11.2 (10–12.1)	5.5 (2.6–7.2)	91 (68–108)	1280 (320–10,240)

*Ø* no reactivity, *BN/AMA+* biochemically normal individuals with positive AMA test, *PBC* primary biliary cholangitis, *ALP* alkaline phosphatase, *ɣGT* gamma glutamyltransfarase, *ELF score* enhanced liver fibrosis score, *PDC-E2* pyruvate dehydrogenase complex E2 subunit, *IIF-AMA* indirect immunofluorescence assays for anti-mitochondria antibodies

^a^Mean and range

^b^Median and range

### AMA and ELF score increase along time in BN/AMA-positive individuals

**Fig. 1:**
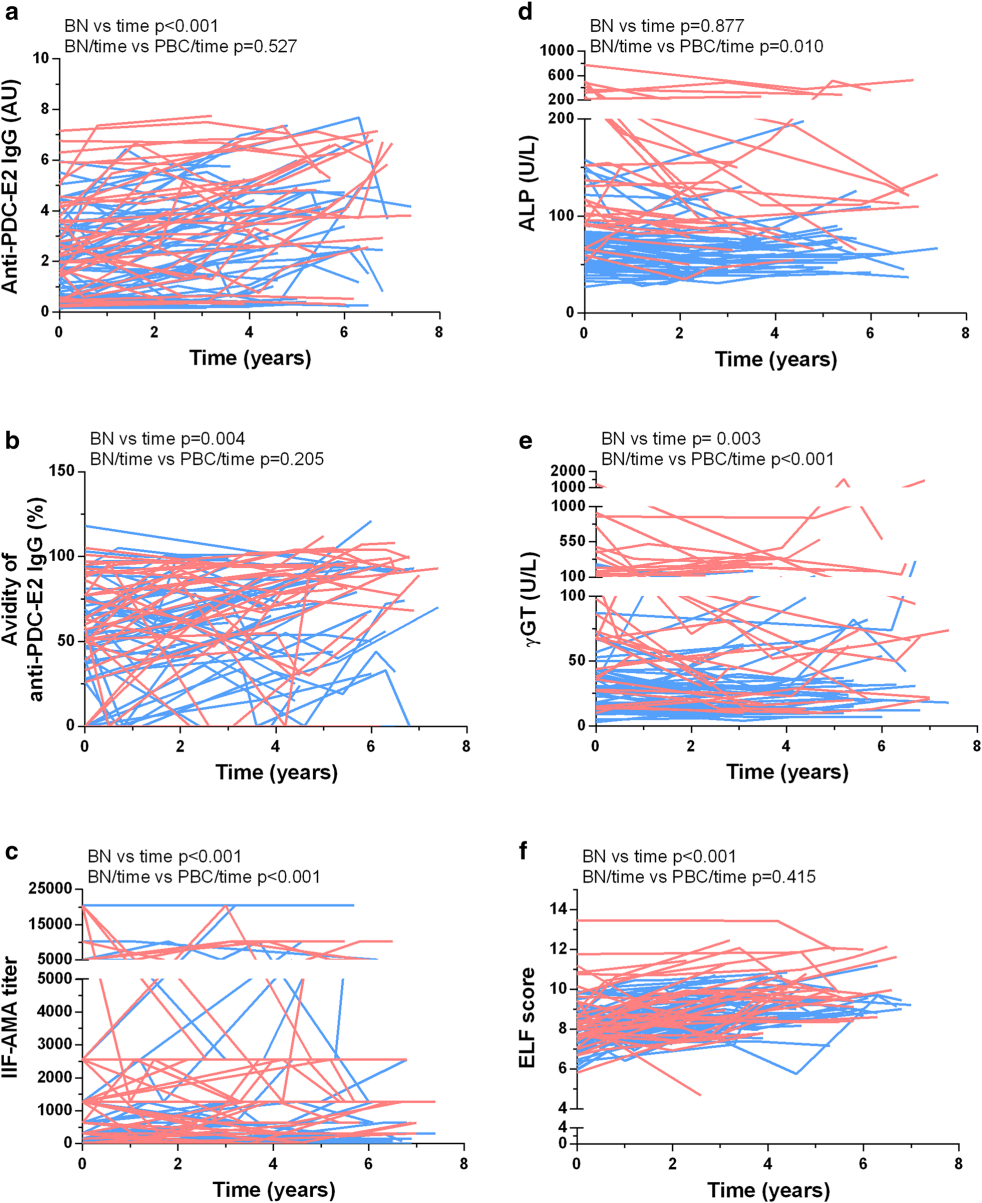
AMA (**a**–**c**) and liver biochemical markers (**d**–**f**) along time in PBC patients and BN/AMA + subjects. BN vs time: temporal change in BN group. *BN/time vs PBC/time* comparison of temporal change in BN/AMA+ subjects versus PBC patients, *BN/AMA+* biochemically normal AMA-positive individuals (BLUE), *PBC* primary biliary cholangitis (RED). Index anti-PDC-E2 IgG: arbitrary units (AU) calculated relative to cut-off

**Fig. 2:**
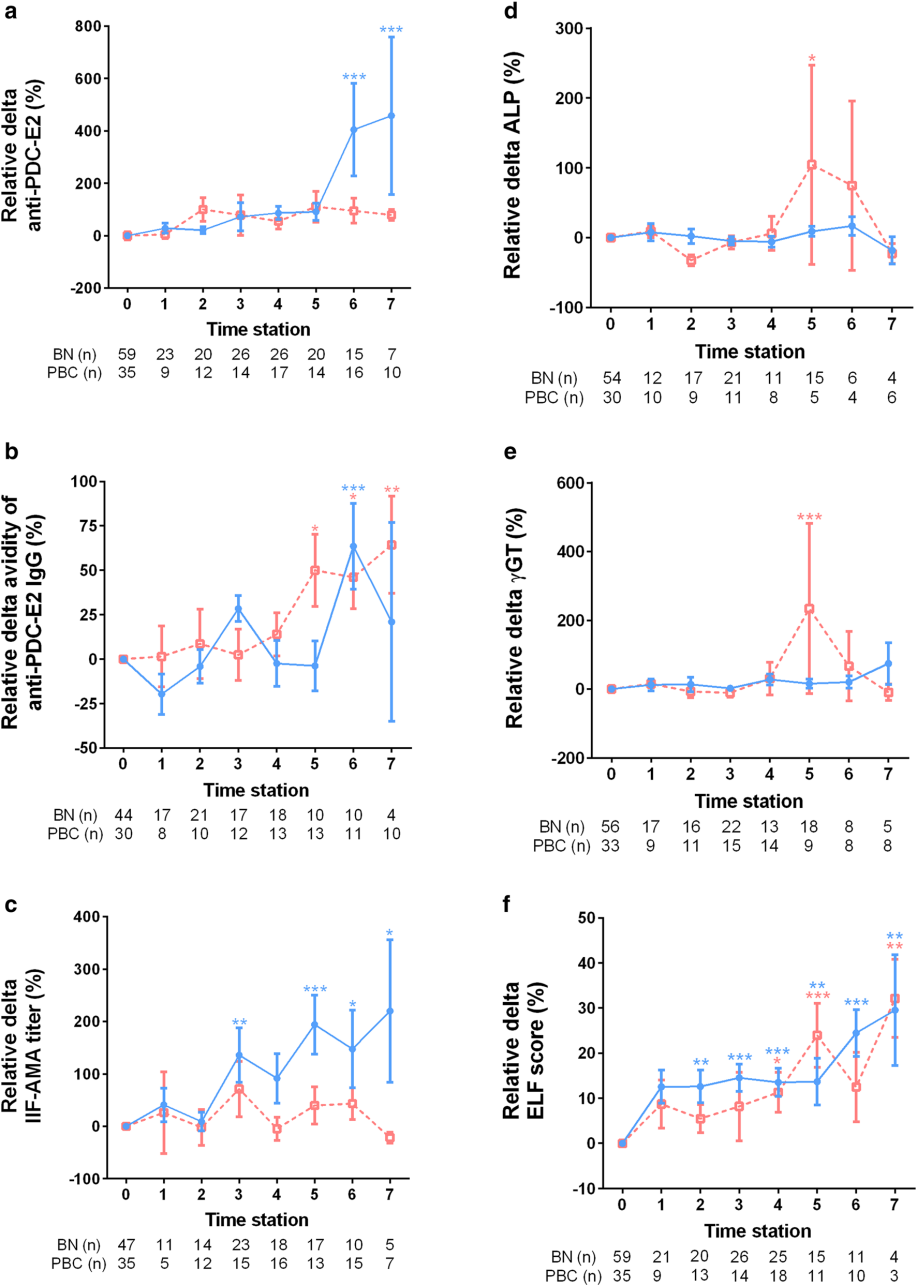
Mean and standard error of relative deltas of AMA (**a**–**c**) and liver biochemical markers (**d**–**f**) at successive time points in BN/AMA-positive individuals and in PBC patients. Relative delta: difference between any time point station and baseline value divided by baseline value. *BN/AMA-positive* biochemically normal AMA-positive individuals (blue solid line), *PBC* primary biliary cholangitis (red dashed line). *p < 0.05; **  < 0.01; ***p < 0.001

Figure 1 depicts ELF score and serum levels of autoantibodies, ɣGT and ALP over time in individual BN/AMA-positive subjects and PBC patients. The consolidated data for each group over the sequential time stations are shown as relative changes to baseline (Fig. 2) for the two groups. There was a significant increase in serum anti-PDC-E2 levels (mean 10.45%/year; p < 0.001), anti-PDC-E2 avidity (mean 3.02%/year; p = 0.004) and ELF score (mean 3.24%/year; p < 0.001) in BN/AMA-positive individuals. A similar increase was observed in PBC patients for anti-PDC-E2 (mean 8.86%/year), anti-PDC-E2 avidity (mean 4.94%/year) and ELF score (mean 2.71%/year), with no significant difference regarding the temporal behavior of BN/AMA subjects (p = 0.527, p = 0.205 and p = 0.415, respectively). Over time, BN/AMA-positive individuals showed an increase in serum IIF-AMA (mean reciprocal titer 6.59%/year; p < 0.001) and ɣGT (mean 2.36%/year; p = 0.003) whereas PBC patients showed a decrease in IIF AMA (mean reciprocal titer − 4.89%/year; p < 0.001) and in ɣGT (mean − 3.88%/year; p < 0.001). ALP decreased over time in PBC patients (mean − 4.83%/year; p = 0.010) and did not change in BN/AMA-positive individuals (p = 0.877).

### The intensity of AMA response correlates with increasing ELF score over time

**Fig. 3:**
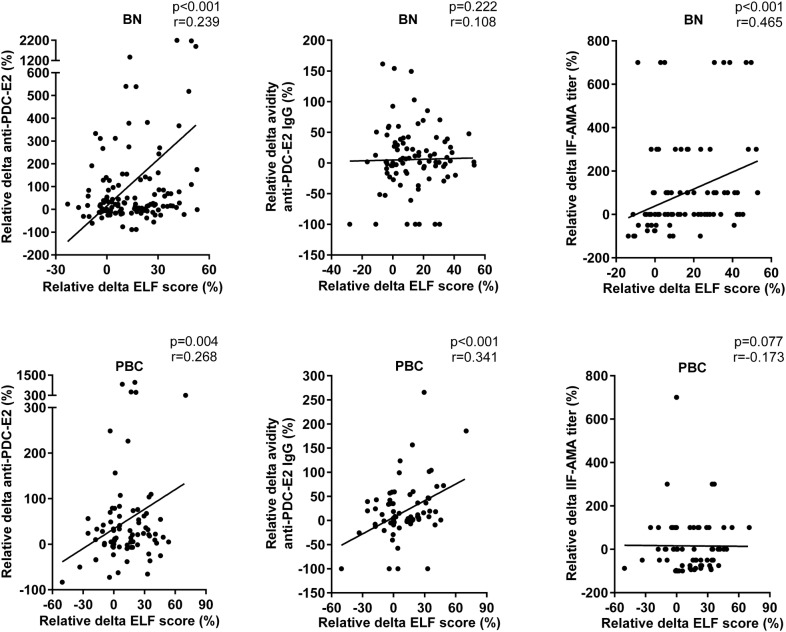
Positive correlation between longitudinal increase in AMA and ELF score. Relative delta: difference between any time point station and baseline value divided by baseline value. Dots represent individuals in each group. The line represents the linear trend regarding the distribution of points

To further investigate the relationship between ELF score and humoral autoimmune response, we compared the temporal change in ELF score and immunological parameters (Fig. 3). In BN/AMA-positive individuals there was modest/low positive correlation of ELF score with IIF-AMA titer (r = 0.465; p < 0.001) and anti-PDC-E2 levels (r = 0.239; p < 0.001). In PBC patients there was low positive correlation of ELF score with anti-PDC-E2 levels (r = 0.268; p = 0.004) and anti-PDC-E2 avidity (r = 0.341; p < 0.001).

### Expansion of autoimmune targets in BN/AMA-positive individuals and PBC patients over time

We evaluated changes in immune responses to the nuclear protein sp100, the nuclear envelope protein gp210, the centromere proteins CENP A/B and to four specific mitochondria antigens compared to T_0_. We observed an increase in the frequency of samples presenting autoantibodies to six of the seven evaluated targets in the BN/AMA-positive group and to four of the seven targets in the PBC group (Table 3). There was a consistent increase in frequency of positive samples for each autoantibody over time. For some autoantibodies, the number of positive responses was too small to allow robust statistical evaluation. Considering the number of recognized antigenic targets in the baseline sample (T_0_) and in the sample in which there was appearance of a new autoantibody (T_X_) for each individual, there was a significant increase in the number of recognized targets over time in BN/AMA-positive individuals (p = 0.034) and in PBC patients (p = 0.024), and the two groups did not differ in this respect (p = 0.242). Over time, at least one additional target was recognized by 39% of BN/AMA-positive individuals and 49% of PBC patients; at least two additional targets by 10% of BN/AMA-positive individuals and 23% of PBC patients; and three or more additional targets by at least 3% of BN/AMA-positive individuals and 3% of PBC patients.

**Table 3:** Expansion of autoantibody antigenic targets related to primary biliary cholangitis along follow-up

Target	Time^a^	Groups			
		BN/AMA+ (n 59)	p	PBC (n 35)	p
Sp100^b^	T_0_	15 (25%)	0.097	4 (11%)	0.048
	T_X_	19 (32%)		10 (29%)	
Gp210^b^	T_0_	4 (7%)	1.000	3 (9%)	0.094
	T_X_	4 (7%)		7 (20%)	
CENP-A/CENP-B^b^	T_0_	0 (0%)	0.317	6 (17%)	0.317
	T_X_	1 (2%)		5 (14%)	
PDC-E2/74 kDa-band^c^	T_0_	42 (71%)	0.053	32 (91%)	0.317
	T_X_	48 (81%)		31 (87%)	
56 kDa-band^c^	T_0_	3 (5%)	0.154	2 (6%)	0.074
	T_X_	5 (8%)		5 (14%)	
52 kDa-band^c^	T_0_	25 (42%)	0.029	21 (60%)	1.000
	T_X_	31 (53%)		21 (60%)	
48 kDa-band^c^	T_0_	6 (10%)	0.010	7 (20%)	0.094
	T_X_	12 (20%)		11 (31%)	

*BN/AMA+* biochemically normal individuals with positive AMA test, *PBC* patients with Primary Biliary Cholangitis

^a^T_0_ is the baseline sample; T_X_ is the sequential sample in which there was appearance of new autoantibody, which was variable among different individuals

^b^Determined by ELISA

^c^Determined by Western blot

In the BN/AMA-positive group, 18 individuals (30.5%) developed antibodies against at least one additional mitochondrial protein, as assessed by WB-AMA, and eight individuals (13.6%) developed autoantibodies to sp100 protein, gp210 protein or CENP-A/B over the course of the study. Among seven individuals reacting only with mitochondria at baseline, six developed anti-sp100 antibodies and one developed anti-sp100 and anti-CENP-A/B antibodies. One individual had AMA and anti-sp100 antibodies at baseline and developed anti-gp210 antibodies.

In the PBC group, 11 patients (31.4%) developed antibodies against at least one additional mitochondrial protein and 12 patients (34.3%) developed autoantibodies to sp100 protein, gp210 protein or CENP-A/B. Of these 12 patients, nine had only AMA reactivity at baseline and developed autoantibodies against other cellular domains (four anti-sp100, two anti-gp210, and three anti-sp100 plus anti-gp210). Two subjects had AMA and anti-CENP-A/B at baseline and developed anti-sp100 antibodies, and one patient had AMA and anti-gp210 at baseline and developed anti-sp100 antibodies.

### BN individuals tended to the PBC-like humoral autoimmune profile over time

**Fig. 4:**
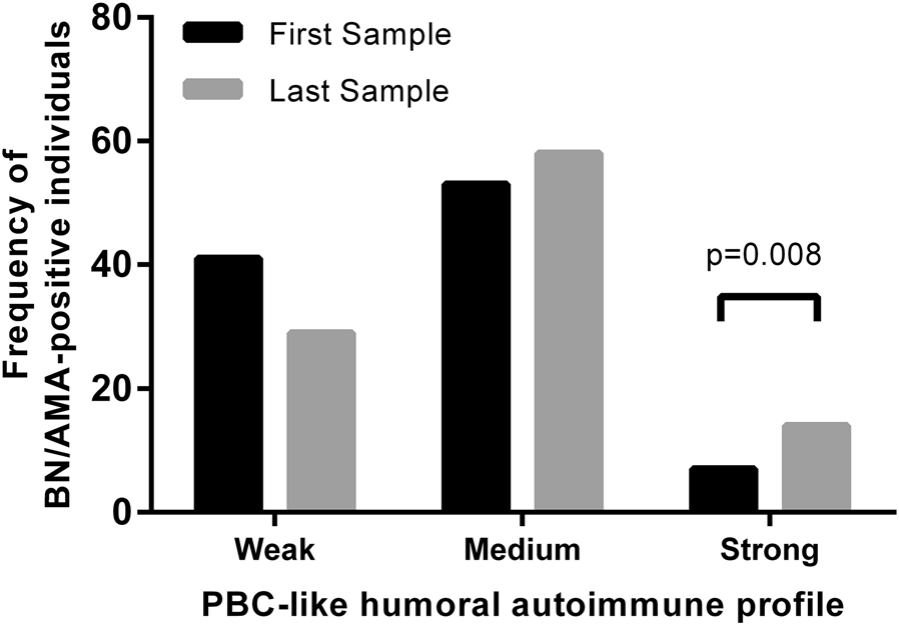
Frequency of BN/AMA-positive individuals with weak (less than 36% of probability), medium (between 36% and 72%) and strong (more than 72%) PBC-like humoral autoimmune profile at baseline and along follow-up. *BN/AMA-positive* subjects with anti-mitochondria antibodies and normal serum liver enzymes

We have previously shown that three autoantibody variables are independently associated with PBC diagnosis in AMA-positive individuals: high avidity anti-PDC-E2 antibodies, high titer IIF-AMA and recognition of three or more cell domains by autoantibodies [16]. We compared these three variables at baseline and at the latest time station for each BN/AMA-positive individual to check for a possible migration towards a PBC-like humoral autoimmune profile [16] over time. The frequency of BN/AMA-positive individuals with humoral autoimmune profile strongly associated with PBC increased from 7% to 14% (p = 0.008) (Fig. 4). Conversely, the frequency of individuals with a humoral autoimmune profile with weak association with established PBC [16] decreased from 41 to 29%. We could not find any relevant peculiarity in the individuals who migrated towards a strong PBC-like humoral autoimmune profile with respect to ELF score and liver enzymes (data not shown).

### Patterns of change in anti-PDC-E2 antibody level and ELF score are heterogeneous among BN/AMA-positive Individuals

**Fig. 5:**
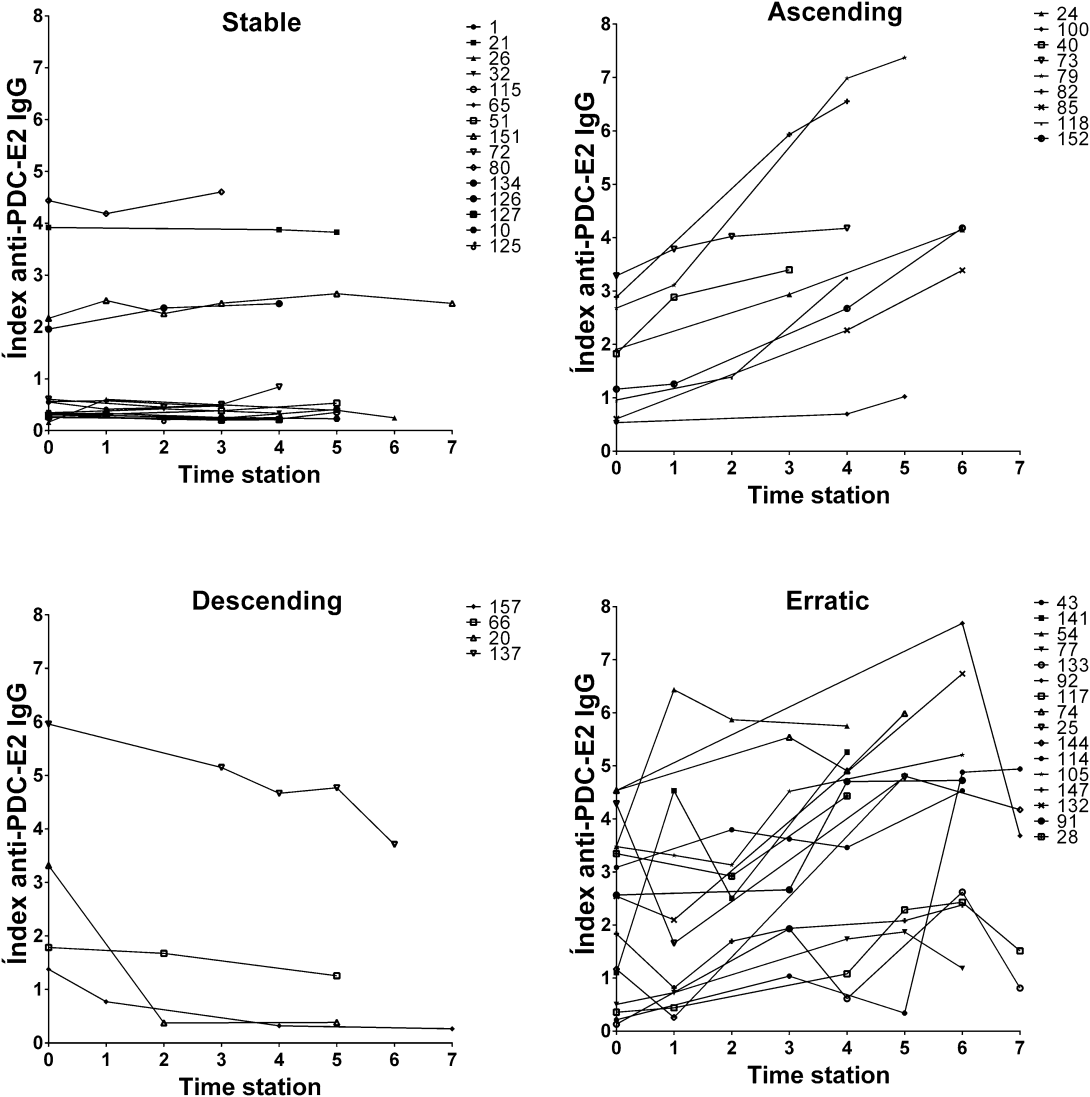
BN/AMA-positive individuals show four patterns of temporal behavior of anti-PDC-E2 antibodies in successive time stations. *BN/AMA-positive* subjects with anti-mitochondria antibodies and normal serum liver enzymes. Index anti-PDC-E2 IgG: arbitrary units (AU) calculated relative to cut-off

**Fig. 6:**
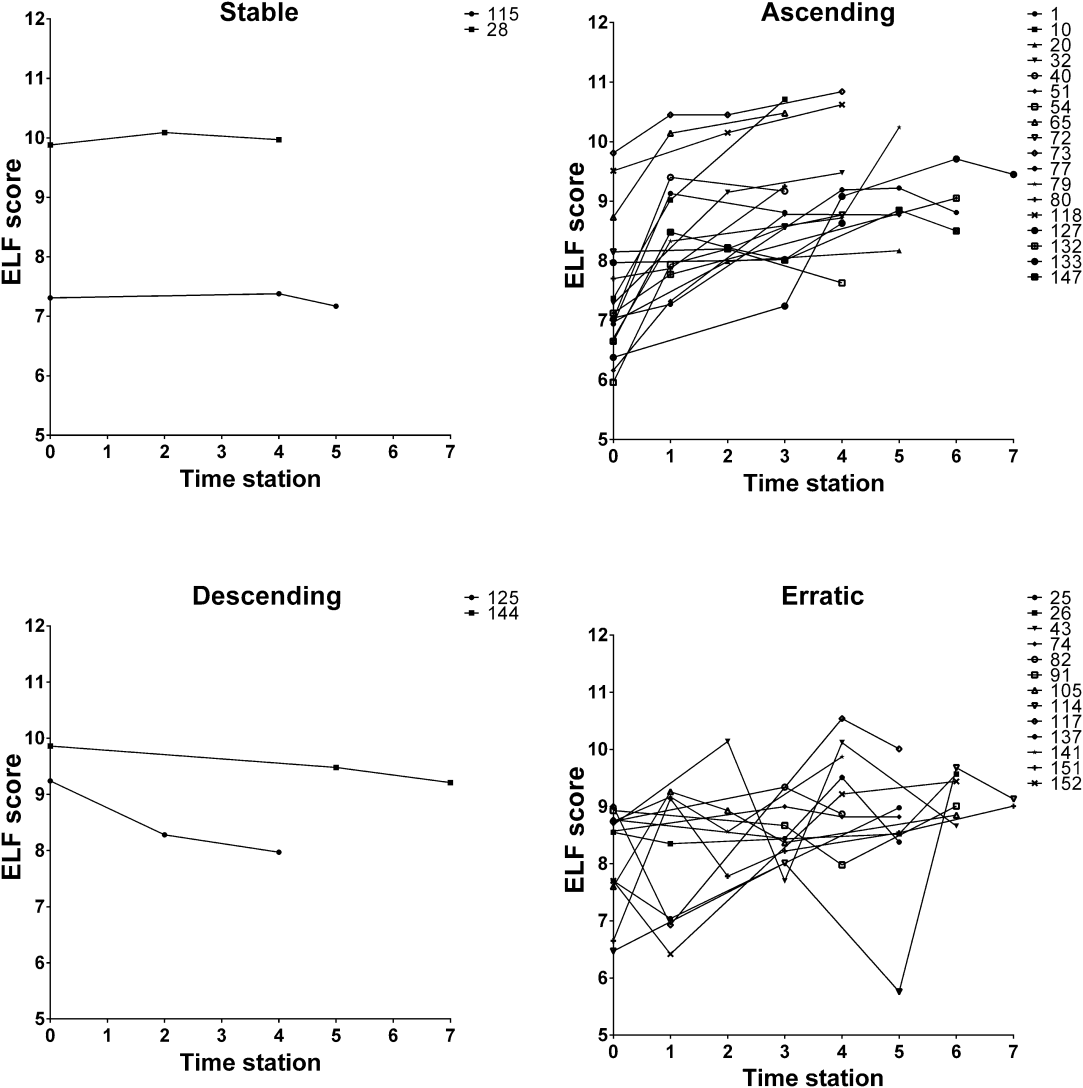
BN/AMA-positive individuals show four patterns of temporal behavior of ELF score in successive time stations. *BN/AMA-positive* subjects with anti-mitochondria antibodies and normal serum liver enzymes

Taking the BN/AMA-positive group as a whole, an overall expansion and intensification of the humoral autoimmune response was observed over time, together with increases in biochemical parameters indicative of hepatic fibrosis. However, the patterns of change for individual members of the group were somewhat heterogeneous. This can be clearly appreciated for anti-PDC-E2 antibody levels and ELF score, for which four divergent longitudinal evolution patterns, tentatively classified as ascending, descending, stable and erratic, were observed (Figs. 5 and 6, respectively).

## Discussion

In this prospective study, we demonstrated intensification of the humoral autoimmune response over time in BN/AMA-positive subjects and PBC patients. Some BN/AMA-positive subjects moved towards a PBC-like humoral autoimmune response profile over the follow-up. As expected, parameters of humoral autoimmune response and liver enzymes were higher in patients with established PBC in comparison to BN/AMA-positive subjects. Of great interest, ELF score, the biochemical biomarker for liver fibrosis, was elevated in many BN/AMA-positive individuals. In addition, ELF score increased progressively over time in BN/AMA-positive subjects and in PBC patients. Remarkably, the increase in ELF score correlated with the enhancement of humoral autoimmune parameters in the BN/AMA-positive group and in PBC patients. Altogether, these findings indicate that even in the absence of clinical symptoms of PBC and traditional liver enzyme abnormalities, some AMA-positive individuals already have evidence of ongoing liver tissue damage. In addition, we provide evidence that there is a potentially progressive process in some of the BN/AMA-positive individuals. The demonstration that some BN/AMA-positive individuals display altered ELF score and continuing intensification of the humoral autoimmune response clearly indicates that these individuals belong to the temporal spectrum of PBC. In such individuals, the incidental identification of AMA offers the opportunity of early diagnosis at pre-clinical stages of the disease and, consequently, the opportunity of early therapeutic intervention.

Several studies have shown that AMA may precede the onset of clinical symptoms and elevated ALP levels for years [29–31]. Mitchison et al. reported on biopsy findings consistent with PBC in AMA-positive asymptomatic individuals without liver enzyme changes [29]. After an average 6-year follow-up of 16 of these individuals, five developed symptoms and 11 showed elevated serum ALP. Kisand et al. followed eight AMA-positive asymptomatic individuals with normal liver enzyme levels for 9 years; three of them showed abnormal elevation of liver enzyme levels on follow-up [30]. Prince et al. estimated that half of the AMA-positive asymptomatic individuals will become symptomatic within a 5-year period, and nearly all patients will develop symptoms over a 20-year period [31]. Lindor et al. estimated that the average time between the first positive AMA test and the establishment of persistently abnormal liver enzyme levels is 6  years, ranging from 1 to 19 years [32]. The results of the present study add to those previous studies by indicating that AMA-positive individuals with normal liver enzymes levels can undergo a progressive and coupled intensification of immunological and liver tissue abnormalities. Therefore, they should be thought of as being potential candidates for early therapeutic intervention at the pre-biochemical or latent phase of PBC [33–36]. As a corollary, such individuals should be actively investigated by biochemical tests, imaging and even biopsy in order to define the actual pathological status of the liver.

The intensification of the humoral autoimmune response in this study comprised increase in AMA titer and avidity, as well as expansion of autoantibody targets. The expansion of the autoantibody profile is a well-known phenomenon in the natural history of autoimmune diseases and involves epitope spreading within the original immunogenic molecule and extension to other polypeptides. We demonstrated expansion of the autoimmune response among polypeptides of the mitochondrial E2 subunit complex and to other polypeptides targeted by autoantibodies in PBC. This observation is a robust indication of enhancement of humoral autoimmune response.

A limitation in the present study is that the exact time point in the natural history of PBC is unknown for the participating BN/AMA-positive individuals. This uncertainty is inherent to the adopted study design and clinical nature of the study. Indeed, PBC is characterized by a heterogeneous natural history that can vary widely among individuals with respect to disease evolution time rate and severity [37]. This limiting factor requires caution and perspective for interpretation of the current findings.

Similarly to what was observed regarding the humoral autoimmune response, ELF score (a biochemical marker for liver fibrosis) and ɣGT (a biochemical marker of liver and biliary damage) increased over time in BN/AMA-positive subjects. This finding indicates a silent progression of liver fibrosis even before increases in enzymes traditionally used as liver damage biomarkers (ALP and ɣGT) are detectable. Indeed, several BN/AMA-positive subjects showed ELF score compatible with moderate fibrosis. This is a crucial finding of the present study, because it is known that early treatment with UDCA can alleviate, and in some cases, prevent the progression of liver injury in patients with clinically established PBC [8, 38, 39]. Therefore, it is reasonable to assume that treatment in preclinical stages might be equally or even more effective.

The occurrence of ELF score compatible with moderate hepatic fibrosis in several BN/AMA-positive subjects indicates that traditional biomarkers of biliary duct involvement (ALP) and hepatocellular damage (aminotransferases) may reflect tissue damage predominantly at relatively advanced stages of the disease. As a corollary, BN/AMA-positive subjects should be considered for more extensive investigation for evidence of ongoing liver inflammation and fibrosis, including ELF score, transient liver elastography, ultrasonography, and magnetic resonance imaging. If these methods indicate potential ongoing pathology, liver biopsy might be indicated.

The observation of progressive intensification of humoral autoimmune response and ELF score prompted us to investigate potential correlation between these parameters. We found that changes in anti-PDC-E2 antibody levels and changes in ELF were correlated in BN/AMA positive subjects and PBC patients. This observation does not allow the inference of a causal relationship, but reinforces the hypothesis that liver damage progression and enhancement of the autoimmune response are related processes associated with PBC primary pathophysiology.

We also observed that not all BN/AMA-positive individuals presented the same pattern of change in immunological and biochemical parameters. Some individuals had frank intensification of humoral autoimmune response, while others showed stable or erratic behavior and still others showed a decrease in some immunological parameters. This observation indicates heterogeneity in the significance of AMA in asymptomatic individuals with normal liver enzyme levels. It is possible that the BN/AMA-positive group comprises not only individuals representing pre-clinical stages of PBC that will eventually develop the expected biochemical and clinical abnormalities, but also individuals that do not belong to the PBC spectrum, in whom AMA represent a spurious finding not related to PBC natural history. This heterogeneity indicates a need for recognition of parameters that specifically identify those that will develop PBC. Non-invasive methods such as liver elastography, magnetic resonance and the ELF score may be a reasonable preliminary approach to identify BN/AMA-positive individuals with possible ongoing liver inflammation and fibrosis processes characteristic of PBC.

## Conclusions

This study revealed a gradual progression in the intensity and scope of the humoral autoimmune response in BN/AMA-positive individuals. This increase was rather consistent, since all analyzed humoral autoimmune parameters (IIF-AMA titer, anti-PDC-E2 levels, anti-PDC-E2 avidity and number of recognized targets) intensified over time. Furthermore, we demonstrate correlation between progression of the humoral autoimmune response and the intensification of biochemical parameters indicative of liver tissue damage in BN/AMA-positive individuals and PBC patients. We also showed that some BN/AMA-positive individuals progressively moved towards a PBC-like humoral autoimmune profile. Finally, we noticed some heterogeneity in the BN/AMA-positive group in that some individuals seem to be clearly in an early stage in PBC natural history whereas others seem not to belong to PBC spectrum. The present findings warrant validation in independent prospective studies over longer periods, as well as controlled studies exploring therapeutic intervention in BN/AMA-positive individuals with subclinical liver tissue inflammation or fibrosis.

## Data Availability

The datasets used and/or analyzed during the current study are available from the corresponding author on reasonable request.

## Abbreviations

PBC: primary biliary cholangitis
AMA: anti-mitochondrial antibodies
UDCA: ursodeoxycholic acid
PDC-E2: anti- pyruvate dehydrogenase complex E2 subunit
ANA: antinuclear antibodies
BN: biochemically normal
ELF: Enhanced Liver Fibrosis
TIMP-1: tissue inhibitor of metallo-proteinases-1
PIIINP: amino-terminal propeptide of type III procollagen
HA: hyaluronic acid
IIF: indirect immunofluorescence assay
ALP: alkaline phosphatase
SISNEP: Brazilian National System of Research Ethics
FITC: fluorescein isothiocyanate
PBS: phosphate-buffered saline pH 7.4
PBS-T: PBS containing 0.05% Tween
PBS-BT: PBS containing 0.05% Tween, 0.5% bovine serum albumin
BSA: bovine serum albumin
HRP: horseradish peroxidase
TMB: 3,3′,5,5′-tetramethylbenzidine
ELISA: Enzyme Linked Immuno Sorbent Assay
2-OADC: 2-oxoacid dehydrogenase complex
BCOADC: Branched-chain 2-Oxo-acid Dehydrogenase
OGDC: 2-Oxo-glutarate Dehydrogenase
WB: Western blot
PBS-M: PBS containing 5% skim milk
CENP: centromere proteins
gp210: anti-glycoprotein-210
sp100: nuclear antigen sp100
ɣGT: gamma-glutamyl transferase
